# The Prothrombotic Phenotypes in Familial Protein C Deficiency Are Differentiated by Computational Modeling of Thrombin Generation

**DOI:** 10.1371/journal.pone.0044378

**Published:** 2012-09-12

**Authors:** Kathleen E. Brummel-Ziedins, Thomas Orfeo, Peter W. Callas, Matthew Gissel, Kenneth G. Mann, Edwin G. Bovill

**Affiliations:** 1 Department of Biochemistry, University of Vermont, College of Medicine, Burlington, Vermont, United States of America; 2 Department of Pathology, University of Vermont, College of Medicine, Burlington, Vermont, United States of America; 3 Department of Mathematics and Statistics, University of Vermont, Burlington, Vermont, United States of America; National Cerebral and Cardiovascular Center, Japan

## Abstract

The underlying cause of thrombosis in a large protein C (PC) deficient Vermont kindred appears to be multicausal and not explained by PC deficiency alone. We evaluated the contribution of coagulation factors to thrombin generation in this population utilizing a mathematical model that incorporates a mechanistic description of the PC pathway. Thrombin generation profiles for each individual were generated with and without the contribution of the PC pathway. Parameters that describe thrombin generation: maximum level (MaxL) and rate (MaxR), their respective times (TMaxL, TMaxR), area under the curve (AUC) and clotting time (CT) were examined in individuals ±PC mutation, ±prothrombin G20210A polymorphism and ±thrombosis history (DVT or PE). This family (n = 364) is shifted towards greater thrombin generation relative to the mean physiologic control. When this family was analyzed with the PC pathway, our results showed that: carriers of the PC mutation (n = 81) had higher MaxL and MaxR and greater AUC (all p<0.001) than non-carriers (n = 283); and individuals with a DVT and/or PE history (n = 13) had higher MaxL (p = 0.005) and greater AUC (p<0.001) than individuals without a thrombosis history (n = 351). These differences were further stratified by gender, with women in all categories generating more thrombin than males. These results show that all individuals within this family with or without PC deficiency have an increased baseline procoagulant potential reflective of increased thrombin generation. In addition, variations within the plasma composition of each individual can further segregate out increased procoagulant phenotypes, with gender-associated plasma compositional differences playing a large role.

## Introduction

Determining who is at risk for thrombotic events is difficult because thrombosis is a multicausal disorder. Venous thromboembolism (VTE) has an annual incidence of >1 per 1,000 person years [1]. VTE mainly consists of deep venous thrombosis (DVT) and its complication, pulmonary embolism (PE). VTE is lethal due mostly to PE [2], which is considered an independent predictor of reduced survival [3]. Methods to reliably identify individuals at risk for VTE would be an important advance.

A genetic risk factor can be detected in approximately 50% of patients with a first episode of VTE [4]. Well-established genetic risk factors for VTE comprise deficiencies or functional abnormalities in two natural anticoagulant pathways: the antithrombin (AT)-heparin sulphate pathway (antithrombin deficiency) and the protein C (PC) pathway, in which protein S (PS) serves as a cofactor (PC deficiency, PS deficiency and resistance to activated PC (APC)) [5], [6], [7], [8]. Another mutation (prothrombin G20210A) has been associated with a 30–70% increase in prothrombin levels and has been weakly correlated with VTE risk [9]. The PC pathway provides a dynamic inhibitory system to regulate thrombin production [10]. If one looks at the prevalence of thrombophilic risk factors, defects in the PC system taken together (PC, PS and fV^Leiden^) are the single most prevalent (28.8%) abnormality [11]. In the EPCOT study [12] of first venous thrombotic events in carriers of familial thrombophilic defects, the majority of first events were associated with abnormalities of components of the PC system. Thus, defects in the PC system are the most prevalent thrombophilic risk factors in thrombophilia. Understanding what occurs in individuals with defects in this pathway may help in understanding potential mechanisms of VTE risk as a multicausal disease.

Homozygous PC deficiency is associated with severe thrombotic tendencies and can result in fatal neonatal thrombotic events [13]. Heterozygous PC deficiency is also associated with an increased risk of thrombosis [14], [15], [16]. The prevalence of PC deficiency is estimated to be 0.5% in the general population [17], [18]. Studies of selected PC deficient families have shown that heterozygous individuals have a 50% chance of experiencing a first venous thromboembolic event by the age of 45 [19], [20], but their overall mortality is not affected [21]. A population with familial type I PC deficiency, first described by Bovill *et al.* [22] identified not only a high incidence of VTE in PC deficient individuals, but also a strong relationship between PC deficiency and venous thrombosis in the family (relative risk = 11.7, p<0.001). About 15% of those with venous thrombosis were not PC deficient. The phenotypic pattern in this family led to the conclusion that they have another genetic risk factor which interacts with PC deficiency to increase the risk of thrombosis [15]. Thus, this family is an ideal cohort for investigating what other hemostatic variables along with PC deficiency might account for thrombosis.

Thrombin has long been recognized for its multiple functions in blood coagulation and platelet aggregation as well as its roles in tissue repair, development and pathogenic processes [23], [24]. Methods that profile thrombin generation, either directly or indirectly, have potential utility in the realm of clinical testing [25], since these methods provide a significant increase in the information collected relative to that available with standard clotting time tests designed for the evaluation of deficiencies in coagulation factors. However, there is great diversity in experimental hematology protocols, resulting in the widely-recognized inter-laboratory variability in the results of thrombin generation studies [26]. One of the approaches to evaluating thrombin generation is to use mathematical models [27], [28], [29], [30], [31], [32], [33], [34]. In addition to several studies from our laboratory [31], [35], [36] presenting empirical validation of our computational model, it was recently evaluated independently against datasets from different laboratories, and showed reasonable agreement with the experimental data [33].

One focus of our group has been to use computational modeling to study the effects that normal range compositional differences in the coagulation proteomes of individuals have on their thrombin generation profiles [36], [37], [38] and test whether differences in predicted thrombin generation segregate with potential risk factors [36], [37], [39], [40]. In this study we evaluated the plasma composition derived thrombin generation profiles from individuals within familial protein C deficiency. To do so, we combined our previously described model of tissue factor initiated thrombin generation [41] with an empirically validated description of the protein C pathway [42]. The potential promoters of thrombotic risk that were evaluated in this family, included the presence of the PC mutation, the presence of the prothrombin G20210A polymorphism, a past history of thrombosis (DVT or PE) and gender.

## Results

### The Variation in Thrombin Generation within this Family

**Figure 1:**
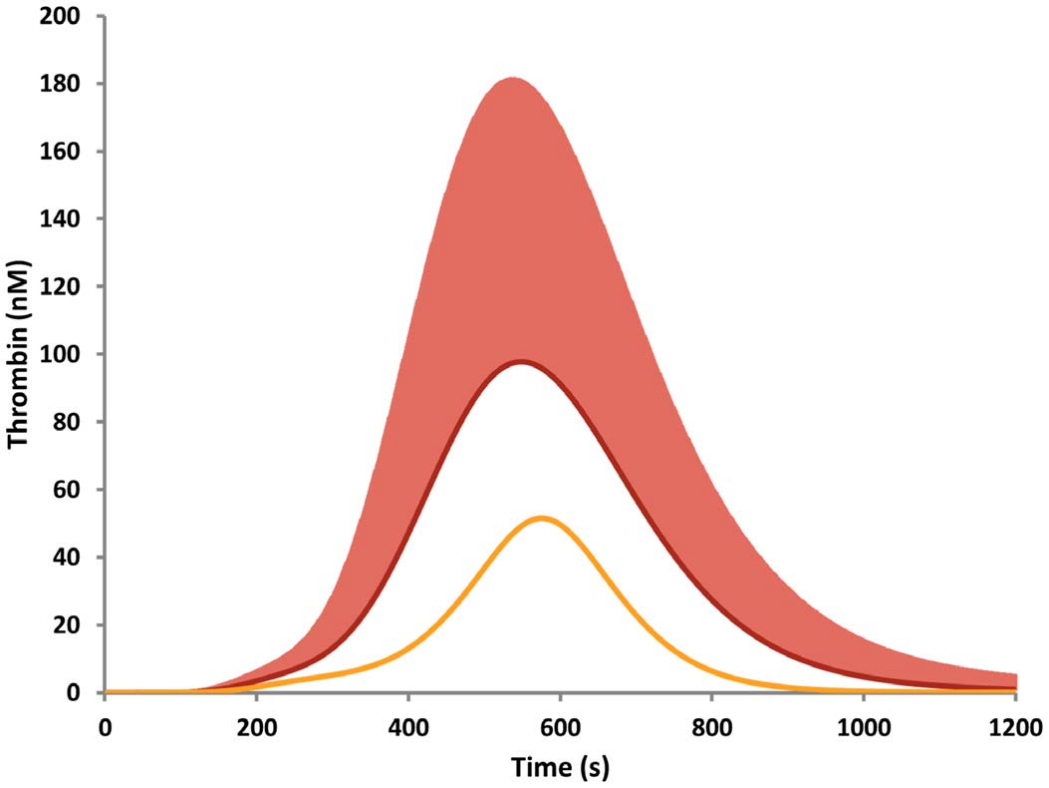
Variation of thrombin generation in familial PC deficiency. Thrombin generation profiles were generated from each individual’s plasma composition (n = 364), containing fII, fV, fVII, fVIII, fIX, fX, AT, TFPI and PC and a 5 pM Tf initiator. The mean thrombin curve is shown in maroon with the standard deviation in salmon. A control curve, representing mean physiologic concentrations of each factor is illustrated in gold as a comparison.

Thrombin generation within this entire family shifted towards greater thrombin generation relative to the mean physiologic control (Figure 1). The MaxR varied from 0.02–2.75 nM/s with a mean of 0.65 (0.52) nM/s. The MaxL varied 78 fold (6–478 nM) with a mean of 112 (88) nM. The AUC varied 83 fold, 3–250 µM·s with a mean of 35 µM·s. The TMaxL varied 2 fold (426–825 s), and the TMaxR varied 3.3 fold (212–716).This large variation of thrombin generation is shown as the standard deviation in grey in Figure 1.

The simulations of thrombin generation are dependent on each individual’s plasma composition. As shown in Table 1, a large variation is observed: specifically fV (8.5 fold), fVIII (7.5 fold) and PC (13 fold).

**Table 1:** Plasma composition within familial PC deficiency.

Protein	Mean (SD)	Range	Physiological mean	Clinically accepted normal range
FII, µM	1.8 (0.4)	0.7–2.9	1.4	0.8–2.0
FV, nM	20.1 (6.7)	7.0–60.0	20.0	12–28
FVII, nM	10.0 (2.5)	4.0–20.5	10.0	6–14
FVIII, nM	0.8 (0.3)	0.24–1.8	0.7	0.4–1.6
FIX, nM	93.3 (27)	47.6–225	90.0	62–135
FX, nM	167 (38)	79.8–274	160	96–224
AT, µM	3.4 (0.6)	1.5–5.6	3.6	3.2–6.3
TFPI, nM	2.3 (0.6)	1.1–5.0	2.5	1.1–4.3
PC, nM	96 (38)	16.0–207	65	50–119

### Investigating the Range of Thrombin Generation within Familial PC Deficiency

#### Effect of PC mutation

**Figure 2:**
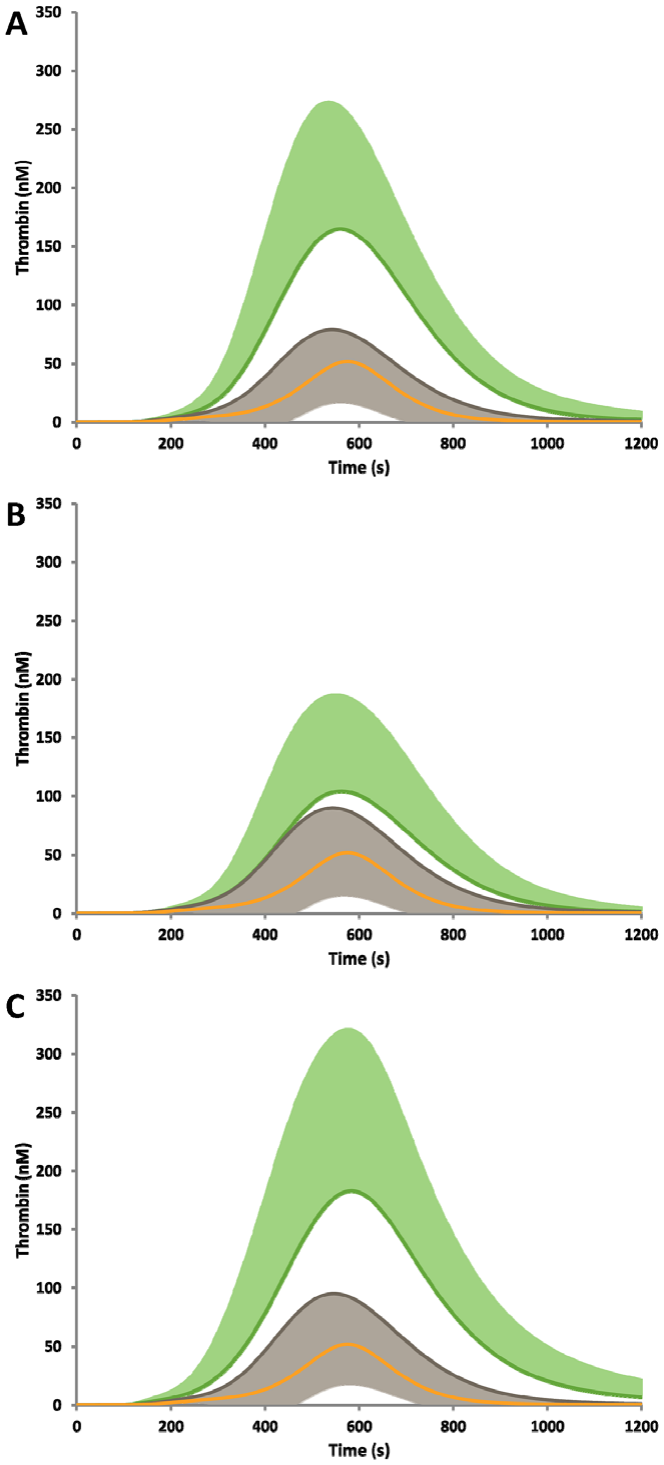
Investigating the range of thrombin generation within familial PC deficiency. Individuals within the family were segregated by **A)** Protein C mutation, **B)** Prothombin G20210A polymorphism, and **C)** Thrombosis history. The groups with the mutation/history are shown in green (+SD) and without the mutation/history are shown in grey (–SD). A control curve, representing mean physiologic concentrations of each factor is illustrated in gold as a comparison.

The contribution of the PC mutation to simulated thrombin generation is shown in Figure 2 panel A and in Table 2. Individuals that are grouped as positive for the PC mutation (n = 81) have greater thrombin generation than individuals within the family that do not have the PC mutation (n = 283). The MaxL, MaxR and AUC were significantly different between the groups. The plasma composition in individuals with the PC mutation had significantly lower levels of fII, fV, fIX, TFPI and PC (Table 3).

**Table 2:** Thrombin generation parameters within groups and stratified by gender.

	All Subjects (Mean (SD))			Females (Mean (SD))			Males (Mean (SD))		
1.PC mutation	Yes N = 81	No N = 283	P value	Yes N = 54	No N = 159	P value	Yes N = 27	No N = 124	P value
Clot Time (s)	313 (93)	340 (93)	0.07	287 (98)	334 (97)	0.005	358 (85)	349 (84)	0.52
Max Rate (nM/s)	1.01 (0.47)	0.55 (0.46)	<0.001	1.15 (0.50)	0.59 (0.50)	<0.001	0.76 (0.41)	0.50 (0.40)	0.003
Max Level (nM)	182 (78)	93 (77)	<0.001	208 (85)	97 (84)	<0.001	135 (65)	86 (64)	0.11
AUC (µM*s)	59 (29)	28 (28)	<0.001	70 (32)	30 (32)	<0.001	41 (21)	25 (21)	0.08
**2.PT mutation**	**Yes N = 43**	**No N = 292**	**P value**	**Yes N = 21**	**No N = 174**	**P value**	**Yes N = 22**	**No N = 118**	**P value**
Clot Time (s)	335 (96)	336 (95)	0.85	291 (101)	327 (101)	0.11	380 (85)	349 (84)	0.14
Max Rate (nM/s)	0.68 (0.47)	0.61 (0.47)	0.47	0.82 (0.52)	0.67 (0.52)	0.23	0.53 (0.39)	0.53 (0.39)	0.92
Max Level (nM)	121 (81)	104 (80)	0.30	142 (91)	114 (91)	0.24	98 (62)	89 (62)	0.61
AUC (µM*s)	39 (30)	32 (30)	0.22	47 (35)	36 (35)	0.25	31 (20)	26 (20)	0.38
**3.Thrombosis**	**Definite N = 13**	**No history N = 351**	**P value**	**Definite N = 8**	**No history N = 205**	**P value**	**Definite N = 5**	**No history N = 146**	**P value**
Clot Time (s)	321 (95)	334 (93)	0.40	292 (101)	323 (99)	0.42	359 (87)	350 (84)	0.81
Max Rate (nM/s)	0.89 (0.51)	0.65 (0.50)	0.11	1.06 (0.56)	0.72 (0.55)	0.14	0.67 (0.43)	0.55 (0.41)	0.53
Max Level (nM)	182 (86)	110 (84)	0.005	226 (96)	122 (95)	0.006	115 (69)	94 (67)	0.49
AUC (µM*s)	67 (31)	34 (30)	<0.001	88 (35)	38 (35)	<0.001	34 (23)	28 (22)	0.56

For all thrombin parameters measured, thrombin generation was greater than the mean physiologic control. The percentage of subjects with the PC mutation for whom the thrombin generation parameters (CT, MaxL, MaxR, AUC) exceeded those for the mean physiologic control ranged between 84% and 95%. Without the PC mutation, this percentage was still 67–70%. The mean PC concentration in the group with the PC mutation was 47 (29) nM. In the group without the PC mutation, the mean concentration of PC was 110 (28) nM. The mean physiologic control has a PC concentration of 65 nM. Thus, the increased thrombin generation within this family is not due to PC alone.

#### Effect of prothrombin G20210A polymorphism

Although the prothrombin concentration was higher in the subjects with this mutation (2.1 (0.4) µM vs. 1.7 (0.4) µM), there were no differences between any thrombin parameters between the two groups (Figure 2 Panel B).

#### Thrombosis history

Individuals within this family, not on any anticoagulants at the time of the blood draw, with a previous history of thrombosis (n = 13) were compared to the larger group without a previous thrombosis history (n = 351). Thrombin generation was greater in this small subset (Figure 2, panel C). Specifically, the MaxL of thrombin was greater (182 (86) nM vs. 110 (84) nM, p = 0.005) and the total amount of thrombin generated (AUC: 67 (31) µM·s vs. 34 (30) µM·s, p = <0.001). Plasma composition within individuals with a previous thrombosis, showed greater fV and fVIII and suppressed PC and fIX (Table 3). These lower levels of PC stem from the fact that 8 out of the 13 individuals within this category had the PC mutation.

**Table 3:** Plasma composition comparison within groups and stratified by gender.

	All Subjects (Mean (SD))			Females (Mean (SD))			Males (Mean (SD))		
1.PC mutation	Yes N = 81	No N = 283	P value	Yes N = 54	No N = 159	P value	Yes N = 27	No N = 124	P value
FII, µM	1.7 (0.4)	1.8 (0.4)	0.01	1.7 (0.4)	1.8 (0.4)	0.05	1.8 (0.4)	1.8 (0.4)	0.75
FV, nM	19.4 (6.8)	20.6 (6.7)	0.05	18.7 (6.5)	19.9 (6.5)	0.18	20.0 (7.0)	21.2 (6.9)	0.42
FVII, nM	9.9 (2.5)	10.1 (2.4)	0.28	10.0 (2.8)	10.3 (2.8)	0.64	9.9 (1.9)	9.8 (1.8)	0.86
FVIII, nM	0.85 (0.27)	0.79 (0.27)	0.13	0.83 (0.25)	0.77 (0.25)	0.12	0.85 (0.29)	0.81 (0.28)	0.52
FIX, nM	88 (27)	94 (26)	0.03	91 (29)	97 (29)	0.26	83 (23)	93 (22)	0.03
FX, nM	160 (39)	169 (38)	0.08	161 (38)	170 (38)	0.22	160 (38)	168 (38)	0.60
AT, µM	3.5 (0.6)	3.3 (0.6)	0.10	3.4 (0.6)	3.3 (0.6)	0.79	3.7 (0.5)	3.3 (0.5)	0.004
TFPI, nM	2.2 (0.6)	2.4 (0.6)	0.04	2.0 (0.6)	2.3 (0.5)	0.005	2.4 (0.6)	2.5 (0.6)	0.59
PC, nM	47 (29)	110 (28)	<0.001	45 (28)	112 (27)	<0.001	51 (29)	108 (29)	<0.001
**2.PT mutation**	**Yes N = 43**	**No N = 292**	**P value**	**Yes N = 21**	**No N = 174**	**P value**	**Yes N = 22**	**No N = 118**	**P value**
FII, µM	2.1 (0.4)	1.7 (0.4)	<0.001	2.1 (0.4)	1.7 (0.4)	<0.001	2.2 (0.4)	1.7 (0.3)	<0.001
FV, nM	20.3 (6.8)	20.4 (6.9)	0.70	19.1 (6.7)	19.8 (6.7)	0.69	21.5 (7.0)	21.1 (6.9)	0.86
FVII, nM	10.3 (2.5)	10.0 (2.5)	0.92	10.2 (2.8)	10.2 (2.8)	0.43	10.3 (1.9)	9.7 (1.8)	0.14
FVIII, nM	0.81 (0.26)	0.80 (0.27)	0.94	0.81 (0.25)	0.77 (0.25)	0.93	0.82 (0.28)	0.82 (0.28)	0.95
FIX, nM	86 (27)	94 (27)	0.14	89 (29)	95 (29)	0.35	84 (23)	92 (22)	0.11
FX, nM	178 (38)	165 (39)	0.15	176 (39)	166 (39)	0.92	180 (38)	164 (38)	0.07
AT, µM	3.3 (0.6)	3.4 (0.6)	0.33	3.1 (0.6)	3.3 (0.6)	0.08	3.4 (0.6)	3.4 (0.6)	0.64
TFPI, nM	2.5 (0.6)	2.3 (0.6)	0.22	2.2 (0.6)	2.2 (0.6)	0.76	2.8 (0.6)	2.4 (0.6)	0.06
PC, nM	91 (37)	100 (38)	0.37	92 (38)	100 (38)	0.33	90 (37)	101 (36)	0.35
**3.Thrombosis history**	**Definite N = 13**	**No history N = 351**	**P value**	**Definite N = 8**	**No history N = 205**	**P value**	**Definite N = 5**	**No history N = 146**	**P value**
FII, µM	2.0 (0.4)	1.8 (0.4)	0.10	2.0 (0.4)	1.8 (0.4)	0.28	2.1 (0.4)	1.8 (0.4)	0.05
FV, nM	25.2 (6.7)	20.1 (6.7)	0.03	27.5 (6.4)	19.3 (6.3)	0.02	19.6 (7.1)	21.1 (6.9)	0.85
FVII, nM	11.1 (2.5)	10.0 (2.5)	0.23	12.4 (2.8)	10.1 (2.8)	0.04	9.1 (1.9)	9.8 (1.8)	0.48
FVIII, nM	1.04 (0.27)	0.80 (0.27)	0.003	1.02 (0.25)	0.78 (0.25)	0.02	1.06 (0.29)	0.81 (0.28)	0.03
FIX, nM	78 (27)	94 (27)	0.04	78 (29)	96 (29)	0.11	81 (23)	91 (22)	0.19
FX, nM	186 (39)	167 (38)	0.18	199 (38)	166 (38)	0.02	165 (39)	167 (37)	0.87
AT, µM	3.3 (0.6)	3.4 (0.6)	0.84	3.1 (0.6)	3.3 (0.6)	0.25	3.7 (0.6)	3.4 (0.6)	0.13
TFPI, nM	2.6 (0.6)	2.3 (0.6)	0.40	2.5 (0.6)	2.2 (0.6)	0.22	2.7 (0.6)	2.5 (0.6)	0.36
PC, nM	69 (39)	97 (38)	0.03	67 (40)	96 (39)	0.10	71 (37)	99 (36)	0.20

### Gender Effect

**Figure 3:**
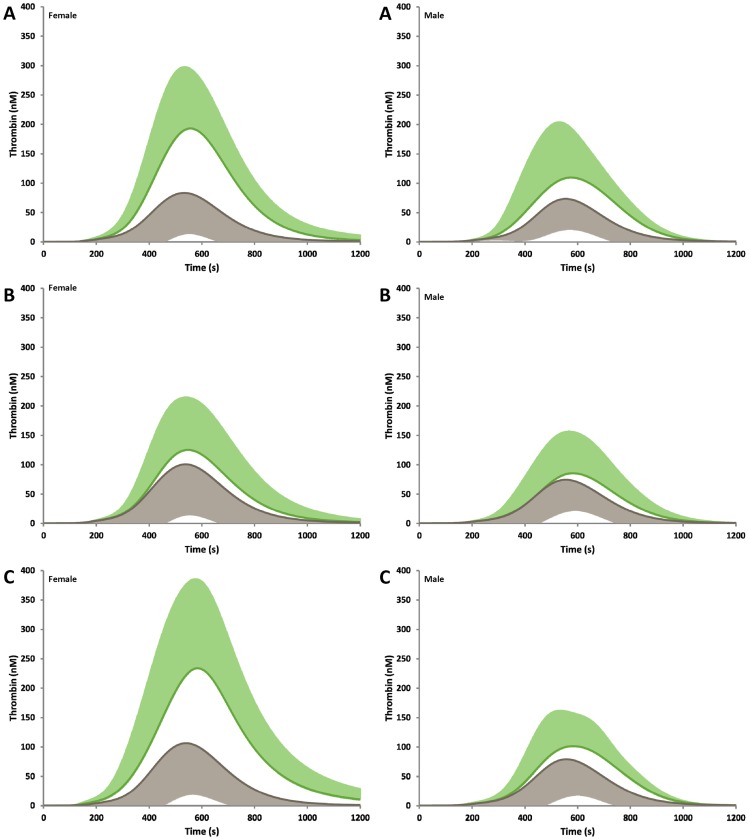
Thrombin generation by gender. Individuals within the family were segregated by gender for **A)** Protein C mutation, **B)** Prothrombin mutation, and **C)** Thrombosis history. The groups with the mutation/history are shown in green (+SD) and without the mutation/history are shown in grey (–SD) A control curve, representing mean physiologic concentrations of each factor is illustrated in gold as a comparison.

Our data was further stratified by gender to determine if there is any relationship between increased thrombin generation in the subsets analyzed. Gender does appear to further segregate women with the PC mutation (Figure 3, panel A and Table 2). Women possessing the PC mutation have a significantly faster clot time and higher maximum rate, maximum level and AUC (all p≤0.005). Surprisingly, only the maximum rate was significantly different in the males when those with and without the PC mutation were compared.

Having a previous history of thrombosis was also further segregated in women (Figure 3, panel C), a higher MaxL (226 (96) nM vs. 122 (95) nM, p = 0.006) and greater AUC (88 (35) µM·s vs. 38 (35) µM·s, p<0.001) were observed.

**Figure 4:**
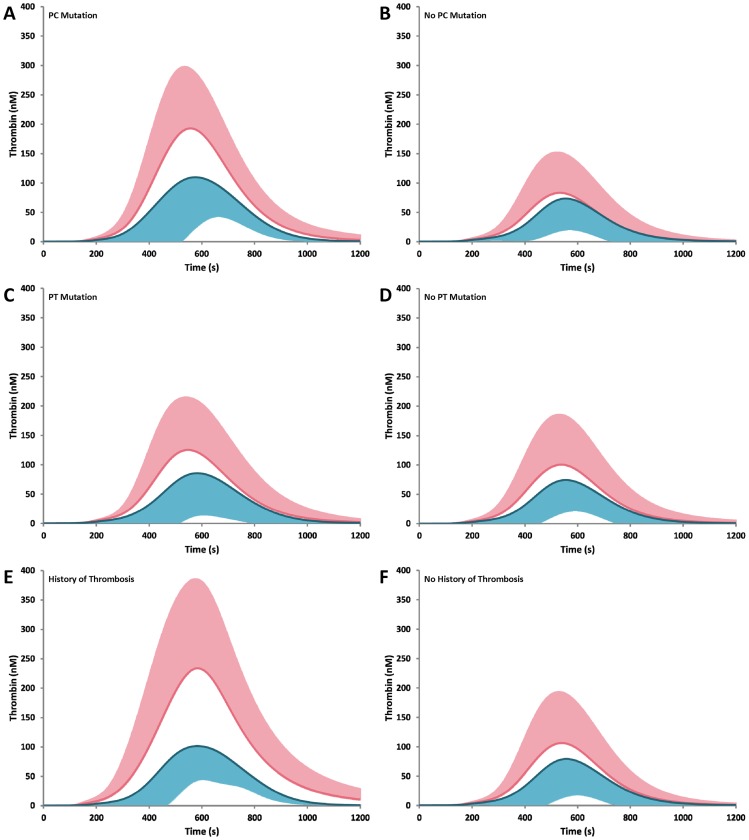
Thrombin generation stratified by gender and additional risk. Males to females with an additional category of risk were compared for either PC mutation (panels A and B), prothrombin G2010A polymorphism (panels C and D), or previous history of thrombosis (panels E and F). Female profiles are shown in pink (+SD). Male profiles are shown in blue (–SD).

In comparing males to females with an additional category of risk (either PC or PT mutation or a previous history of thrombosis, Figure 4), thrombin generation parameters in nearly all cases trend higher in women. This is most notably seen in the PC mutation group (Figure 4, panels E and F), where all parameters are significantly different.

#### Plasma factor compositional differences by gender

Gender plasma compositional differences were seen in all cases evaluated (Table 3). Higher levels of fV, fVII, fVIII, and fX were seen in women with a thrombosis history versus without, and with men only increases in fII and fVIII were seen in men with a previous history of thrombosis.

In women with the PC mutation versus women without, fII, TFPI and PC were significantly lower. Whereas in men with the mutation versus without, fIX and PC were significantly lower and AT was significantly higher. In women with the prothrombin G20201A mutation versus women without, only fII was significantly elevated. This phenomenon was also observed in men.

## Discussion

In this study we show that thrombin generation derived via each individual’s concentrations of pro- and anticoagulant factors identifies groups within familial PC deficiency and in comparison to unrelated controls. Individuals within the family containing risk factors that include the PC mutation, the prothrombin G20210A mutation and having a past history of thrombosis show increased thrombin generation. Females who have any of the evaluated risk factors generate more thrombin than males with the same risk factors. These studies suggest that within this family gender might further influence the risk of thrombosis.

The PC anticoagulant pathway plays a major role in the balance of procoagulation and anticoagulation, by providing a dynamic inhibitory system to regulate thrombin production. APC produced by the thrombin–thrombomodulin complex, inactivates the cofactors fVa and fVIIIa [43], thereby down-regulating further generation of thrombin and stopping clot propagation. In PC deficiency, the down-regulation of thrombin production is compromised. Therefore, thrombin generation as a measure of a thrombosis potential is a good marker in evaluating familial PC deficiency. In a previous study of this family, the genetic basis for coagulation factor hereditability was evaluated and the results showed that the heritability correlated best with measures of thrombin activity [44]. In our current study, we show that by using each individual’s plasma composition to simulate thrombin generation through the relevant PC pathway, thrombin production is elevated in all members of the PC family. This study is the first to show the contribution of the PC pathway, as modeled by each individual’s coagulation factor composition, to thrombin generation in familial PC deficiency.

This family has been extensively studied, and the observed phenotypic pattern led to the conclusion that they have another genetic risk factor which interacts with PC deficiency to increase the risk of thrombosis [15], [45]. Several candidates for the interacting factor have been ruled out [46], including the prostaglandin H synthase 1 gene [47] and platelet-activating factor acetyl-hydrolase Ib [48]. The G20210A prothrombin polymorphism was not found to be associated with risk of venous thrombosis in the family [49], although, in this current study we show that individuals that possess the prothrombin mutation have increased thrombin generation. Factor V^Leiden^ is rare in the family (<2% affected) and thus cannot explain the observed inheritance pattern. Recent genotyping and resequencing have provided some promising evidence of a possible interacting gene, cell adhesion molecule 1 [50]. In this current study, using each individual’s plasma composition to evaluate thrombin generation, we are able to identify that the increased thrombin generation in this family is also not directly related to their level of PC. For example, individuals without the PC mutation display greater thrombin generation despite the fact that their mean PC level was significantly greater than the mean physiologic value. If PC was the only contributing factor, at increasing PC concentrations, thrombin generation would be suppressed.

Consistent with our prior studies [37], [51], our current findings using a computational model which includes a PC pathway component suggest that it is not one factor alone that contributes to thrombin generation dynamics in this subject group, but a combination of each individuals’ other plasma composition factors (as seen in Table 1). If individuals possess higher normal procoagulant and anticoagulant factors, they will generate thrombin faster than individuals with the same PC levels at lower normal procoagulant and anticoagulant levels. Thus, an individual could potentially be at a better hemostatic advantage over another even though the PC levels are equivalent.

In our study, we identify that gender appears to play a large role in this family in that women have increased thrombin generation over men. As well, plasma composition differences were identified in segregating women from men. Previously, we have shown that simulated thrombin generation was increased in healthy women, women with a DVT [37] and women on oral contraceptives [36], [46], [52]. A recent study by Christiansen *et al.* [53] showed that approximately half of the thrombotic recurrences in women were provoked and were mainly related to oral contraceptive use. In earlier research on this family, we found that PC deficiency increased risk of thrombosis in female family members when taking oral contraceptives and during pregnancy [15], [52] Because of those studies, women in the family who are PC deficient were strongly advised against use of oral contraceptives and are almost always given prophylactic heparin during pregnancy. Further studies regarding plasma compositional differences to elucidate the mechanism behind the increased thrombin generation in women and the effect from additional thrombotic risk factors are warranted in this family.

Although, sex differences in thrombosis have been described previously [54], [55], [56], their underlying mechanisms are not completely understood. Since our study involves changes in plasma composition (gender dependent) and increased procoagulant potential, one link between these two (coagulation factors and gender) can be the liver. Coagulation proteins are synthesized in the liver, and liver gene expression is sex specific and depends on sex differences in growth hormone secretion. A study by Wong *et al*. [57] proposed a novel mechanism whereby sex specific growth hormone patterns mediate sex differences in thrombosis through coordinated changes in the expression of coagulation inhibitor genes in the liver. It has also recently been suggested by Tripodi and Mannucci [58] that changes in the balance of pro and anticoagulants in chronic liver disease account for their coagulopathic state. Therefore, changes in liver function in relationship to gender and deficiency state should be further investigated in this family.

## Materials and Methods

Participation of all individuals within the familial PC family was approved by the University of Vermont Human Studies Committee. All participants gave informed written consent.

### Subjects

Our study population is a family with a history of high incidence of VTE (Kindred Vermont II) which was discovered to be PC deficient in the 1980s [22]. The cause of PC deficiency was determined to be a 3363 inserted C mutation in exon 6 of the PC gene [59]. Blood was drawn from 514 members of the extended family. Of family members, 33% are PC deficient and 9% have a verified history of deep vein thrombosis and/or pulmonary embolism. Of the 514 family members drawn, 364 (71%) were included in the current analysis. Reasons for exclusion included: were on coumadin at the time blood drawing (n = 35); unknown coumadin status at the time of blood drawing (n = 5); self-reported but unconfirmed history of DVT or PE (n = 11); history of superficial venous thrombosis but not DVT or PE (n = 18); insufficient sample for analysis (n = 6); and insufficient composition data (n = 75).

### Plasma Composition Analyses

The blood collection procedure and the measurements of the levels of the coagulation proteins fII, fVII, fV, fVIII, fIX, fX, tissue factor pathway inhibitor (TFPI) and antithrombin (AT) from citrated plasma were described in detail in earlier studies within this family [44]. In brief, fII were measured by in-house-developed sandwich-type enzyme linked immunosorbent assays and fV was performed using a commercial assay (Enzyme Research Laboratories, South Bend, IN, USA). The fVII, fVIII, fIX, fX and total TFPI antigen levels were measured by sandwich ELISAs using commercial kits (Assaerachrom, Diagnostica Stago, Parsippany, NJ, USA). PC was measured as an activity assay. The mean (SD) of each of the factor levels are shown in Table 1.

### Numerical Simulations

Our numerical model of the extrinsic coagulation system [41], [60], [61] provides a platform for investigating thrombin generation profiles and patterns in a large group of individuals. In this study, we are incorporating a module of equations describing the protein C pathway. This description primarily derives from a recently published study [42] combining empirical and computational analysis of central elements of this pathway. The complete model (Tables S1, S2,S3) also includes thrombomodulin (Tm) binding to thrombin and meizothrombin and the activation of PC by these complexes [62], [63] and AT inhibition of thrombin-soluble thrombomodulin complexes. The computational inputs included: actual factor levels from each individual in the PC family (n = 364) for fII, fV, fVII, fVIII, fIX, fX, AT, TFPI and PC that were translated into molar concentrations using the mean plasma concentration as 100% and a 5 pM tissue factor (Tf) trigger to correlate with our empirical studies [31], [64]. Thrombomodulin was modeled at 1 nM which is an estimate of the concentration that would be found in medium veins and muscular arteries [65]. These estimates are however, completely based on the diameter of the vessel and the assumption of uniform levels of thrombomodulin expression on endothelial cells throughout the vasculature.

Total active thrombin was simulated at 1 s intervals over 20 minutes and the output was evaluated using the thrombin parameters (maximum level (MaxL) and rate (MaxR) and the corresponding times (TMaxL and TMaxR, respectively) and area under the curve (AUC)). Clot time (CT) was taken to be the time at which 10 nM thrombin is generated [64]. A mean physiologic control was used that sets all the factor levels at mean physiologic concentrations.

### Statistics

Thrombin generation parameters were compared using variance component analysis methods described by Almasy and Blangero [66]. In this approach, models are compared using likelihood-ratio tests, with relatedness of study subjects accounted for as polygenic heritability. In our analysis age and sex were adjusted for by including them as covariates in the models.

## Supporting Information

Table S1 Reaction mechanism of the computational model (list of equations). For equilibrium expressions denoted by ←1–2→, the first number listed describes the reverse/dissociation reaction (k_off_), the second number listed describes the association reaction (k_on_). Notation and the accompanying rate constants are listed in separate tables beneath the list of equations. Complexes are represented with an equal sign between the components. Active enzymes are listed as the zymogen followed by an “a”.
(DOC)

Table S2 Abbreviations used in the computational model.
(DOC)

Table S3 Rate constants used in the computational model.
(DOC)
