# Supplementary material for: The Prothrombotic Phenotypes in Familial Protein C Deficiency Are Differentiated by Computational Modeling of Thrombin Generation
Source: PLoS One. 2012 Sep 12;7(9):e44378. doi: 10.1371/journal.pone.0044378 (PMC3440432; doi:10.1371/journal.pone.0044378)
Supplement: Table S1 — Reaction mechanism of the computational model (list of equations). For equilibrium expressions denoted by ←1–2→, the first number listed describes the reverse/dissociation reaction (koff), the second number listed describes the association reaction (kon). Notation and the accompanying rate constants are listed in separate tables beneath the list of equations. Complexes are represented with an equal sign between the components. Active enzymes are listed as the zymogen followed by an “a”. (DOC) [file pone.0044378.s001.doc]

TF+VII ←1-2→ TF=VII

TF+VIIa ←3-4→ TF=VIIa

TF=VIIa+VII -5→ TF=VIIa+VIIa

Xa+VII -6→ Xa+VIIa

IIa+VII -7→ IIa+VIIa

TF=VIIa+X ←8-9→TF=VIIa=X

TF=VIIa=X -10→ TF=VIIa=Xa

TF=VIIa+Xa ←11-12→ TF=VIIa=Xa

TF=VIIa+IX ←13-14→ TF=VIIa=IX

TF=VIIa=IX -15→ TF=VIIa+IXa

Xa+II -16→ Xa+IIa

IIa+VIII -17→ IIa+VIIIa

VIIIa+IXa ←18-19→ IXa=VIIIa

IXa=VIIIa+X ←20-21→ IXa=VIIIa=X

IXa=VIIIa=X -22→ IXa=VIIIa+Xa

VIIIa ←23-24→ VIII.lca1+VIII.a2

IXa=VIIIa=X -25→ VIII.lca1+VIII.a2+X+IXa

IXa=VIIIa -25→ VIII.lca1+VIII.a2+IXa

IIa+V -26→ IIa+Va

Xa+Va ←27-28→ Xa=Va

Xa=Va+II ←29-30→ Xa=Va=II

Xa=Va=II -31→ Xa=Va+mIIa

mIIa+Xa=Va -32 → IIa+Xa=Va

Xa+TFPI ←33-34→ Xa=TFPI

TF=VIIa=Xa+TFPI ←35-36→ TF=VIIa=Xa=TFPI

TF=VIIa+Xa=TFPI -37→ TF=VIIa=Xa=TFPI

Xa+ATIII -38→ Xa=ATIII

mIIa+ATIII -39→ mIIa=ATIII

IXa+ATIII -40→IXa=ATIII

IIa+ATIII -41→IIa=ATIII

TF=VIIa+ATIII -42→TF=VIIa=ATIII

TM+IIa ←43-44→ TM=IIa

TM=IIa+PC ←45-46→ TM=IIa=PC

TM=IIa=PC -47→ TM=IIa+APC

TM=IIa+ATIII -48→ IIa=ATIII+TM

APC+Va ←49-50→ APC=Va

APC=Va -51→ APC+Va5

APC=Va -52→ APC+Va3

APC+Va5 ←49-50→ APC=Va5

APC+Va3 ←49-50→ APC=Va3

APC=Va3 -51→ APC+Va53

APC=Va5 -52→ APC+Va53

Va3 -53→ HCF+LCA1

Va53 -53→ HCF+LCA1

APC+LCA1 ←49-50→ APC=LCA1

APC+TM=IIa ←45-46→ TM=IIa=APC

Xa+Va5 ←54-28→ Xa=Va5

Xa+Va3 ←54-28→ Xa=Va3

Xa=Va5+II ←29-30→ Xa=Va5=II

Xa=Va5=II -55→ Xa=Va5+mIIa

Xa=Va3+II ←29-30→ Xa=Va3=II

Xa=Va3=II -56→ Xa=Va3+mIIa

Xa=Va5+mIIa -57→ IIa+Xa=Va5

Xa=Va3+mIIa -58→ IIa+Xa=Va3

Xa=Va3 -59→ HCF+LCA1+Xa

Xa=Va3=II -59→ HCF+LCA1+Xa+II

IXa+X -60→ IXa+Xa

mIIa+V -61→ mIIa+Va

TM+mIIa ←43-44→ TM=mIIa

TM=mIIa+PC ←45-46→ TM=mIIa=PC

TM=mIIa=PC -47→ TM=mIIa+APC

TM=mIIa+ATIII -48→ mIIa=ATIII+TM

Xa+Va53 ←54-28→ Xa=Va53

Xa=Va53+II ←29-30→ Xa=Va53=II

Xa=Va53=II -56→ Xa=Va53+mIIa

Xa=Va53+mIIa -58→ IIa+Xa=Va53

Xa=Va53 -59→ HCF+LCA1+Xa

Xa=Va53=II -59→ HCF+LCA1+Xa+II

II+Va ←62-63→ II=Va

Xa=Va5+APC -64→ Xa=Va53+APC
