# Supplementary material for: The Prothrombotic Phenotypes in Familial Protein C Deficiency Are Differentiated by Computational Modeling of Thrombin Generation
Source: PLoS One. 2012 Sep 12;7(9):e44378. doi: 10.1371/journal.pone.0044378 (PMC3440432; doi:10.1371/journal.pone.0044378)
Supplement: Table S2 — Abbreviations used in the computational model. (DOC) [file pone.0044378.s002.doc]

| **Notation** | **Species** |
| --- | --- |
| TF | tissue factor |
| VII | factor VII |
| X | factor X |
| II | prothrombin |
| IX | factor IX |
| VIII | factor VIII |
| VIII.lca1 and VIII.a2 | inactive fragments of factor VIIIa |
| V | factor V |
| mIIa | meizothrombin |
| TFPI | tissue factor pathway inhibitor |
| ATIII | antithrombin-III |
| PC | protein C |
| APC | activated protein C |
| Va5, Va3 and Va53 | partially proteolyzed factor Va  (cleavage at Arg506, Arg306, or both Arg506 and Arg306 respectively) |
| HCF and LCA1 | inactive fragments of factor Va |
| TM | thrombomodulin |
