# Supplementary material for: The Prothrombotic Phenotypes in Familial Protein C Deficiency Are Differentiated by Computational Modeling of Thrombin Generation
Source: PLoS One. 2012 Sep 12;7(9):e44378. doi: 10.1371/journal.pone.0044378 (PMC3440432; doi:10.1371/journal.pone.0044378)
Supplement: Table S3 — Rate constants used in the computational model. (DOC) [file pone.0044378.s003.doc]

| **Reaction #** | **RC (s^-1^)** | **RC (mol^-1^ * s^-1^)** |
| --- | --- | --- |
| 1 | 3.10 * 10^-3^ |  |
| 2 |  | 3.20 * 10^6^ |
| 3 | 3.10 * 10^-3^ |  |
| 4 |  | 2.30 * 10^7^ |
| 5 |  | 4.40 * 10^5^ |
| 6 |  | 1.30 * 10^7^ |
| 7 |  | 2.30 * 10^4^ |
| 8 | 1.05 |  |
| 9 |  | 2.50 * 10^7^ |
| 10 | 6 |  |
| 11 | 19 |  |
| 12 |  | 2.20 * 10^7^ |
| 13 | 2.4 |  |
| 14 |  | 1.00 * 10^7^ |
| 15 | 1.8 |  |
| 16 |  | 7.50 * 10^3^ |
| 17 |  | 2.00 * 10^7^ |
| 18 | 5.00 * 10^-3^ |  |
| 19 |  | 1.00 * 10^7^ |
| 20 | 1.00 * 10^-3^ |  |
| 21 |  | 1.00 * 10^8^ |
| 22 | 8.2 |  |
| 23 |  | 2.20 * 10^4^ |
| 24 | 6.00 * 10^-3^ |  |
| 25 | 1.00 * 10^-3^ |  |
| 26 |  | 2.00 * 10^7^ |
| 27 | 0.075 |  |
| 28 |  | 1.50 * 10^8^ |
| 29 | 103 |  |
| 30 |  | 1.00 * 10^8^ |
| 31 | 63.5 |  |
| 32 |  | 2.30 * 10^8^ |
| 33 | 3.60 * 10^-4^ |  |
| 34 |  | 9.00 * 10^5^ |
| 35 | 1.10 * 10^-4^ |  |
| 36 |  | 3.20 * 10^8^ |
| 37 |  | 5.00 * 10^7^ |
| 38 |  | 4.20 * 10^3^ |
| 39 |  | 7.10 * 10^3^ |
| 40 |  | 4.90 * 10^2^ |
| 41 |  | 7.10 * 10^3^ |
| 42 |  | 2.30 * 10^2^ |
| 43 | 0.33 |  |
| 44 |  | 1.00 * 10^8^ |
| 45 | 100 |  |
| 46 |  | 1.00 * 10^8^ |
| 47 | 0.41 |  |
| 48 |  | 7.10 * 10^3^ |
| 49 | 0.7 |  |
| 50 |  | 1.00 * 10^8^ |
| 51 | 1 |  |
| 52 | 0.192 |  |
| 53 | 0.028 |  |
| 54 | 0.15 |  |
| 55 | 10.3 |  |
| 56 | 10.3 |  |
| 57 |  | 4.60 * 10^7^ |
| 58 |  | 4.60 * 10^7^ |
| 59 | 0.0035 |  |
| 60 |  | 5.70 * 10^3^ |
| 61 |  | 3.00 * 10^6^ |
| 62 | 70 |  |
| 63 |  | 1.00 * 10^8^ |
| 64 |  | 4.05 * 10^6^ |

RC – rate constant. Equations 1–42 are from Hockin *et al*. (ref. 41). Equations 43–48 are from Doyle *et al*. and Cote *et al*. (refs. 62 and 63). Equations 49–59 and 62–64 are from Bravo *et al*. (ref. 42). Equation 60 is from Butenas *et al*. (ref. 61). Equation 61 is from Orfeo *et al*. (ref. 60).
